# Perceived neighborhood environment and physical activity in 11 countries: Do associations differ by country?

**DOI:** 10.1186/1479-5868-10-57

**Published:** 2013-05-14

**Authors:** Ding Ding, Marc A Adams, James F Sallis, Gregory J Norman, Melbourn F Hovell, Christina D Chambers, C Richard Hofstetter, Heather R Bowles, Maria Hagströmer, Cora L Craig, Luis Fernando Gomez, Ilse De Bourdeaudhuij, Duncan J Macfarlane, Barbara E Ainsworth, Patrick Bergman, Fiona C Bull, Harriette Carr, Lena Klasson-Heggebo, Shigeru Inoue, Norio Murase, Sandra Matsudo, Victor Matsudo, Grant McLean, Michael Sjöström, Heidi Tomten, Johan Lefevre, Vida Volbekiene, Adrian E Bauman

**Affiliations:** 1 Department of Family Preventive Medicine, University of California San Diego, La Jolla, California, USA; 2 Graduate School of Public Health, San Diego State University, San Diego, California, USA; 3 Faculty of Medicine, Sydney School of Public Health, University of Sydney, Camperdown, New South Wales, Australia; 4 School of Nutrition and Health Promotion, Arizona State University, Phoenix, ArizonaUSA; 5 Risk Factor Monitoring and Methods Branch, Applied Research Program, National Cancer Institute, Bethesda, Maryland, USA; 6 Division of Physiotherapy, Karolinska Institute, Stockholm, Sweden; 7 Department of Neurobiology Care Sciences and Society, Karolinska Institute, Stockholm, Sweden; 8 Canadian Fitness and Lifestyle Research Institute, Ottawa, Canada; 9 Pontificia Universidad Javeriana, Bogotá, Colombia; 10 Department of Movement and Sport Sciences, Ghent University, Ghent, Belgium; 11 Institute of Human Performance, University of Hong Kong (Macfarlane), Hong Kong, China; 12 School of Nutrition and Health Promotion, Arizona State University, Phoenix, Arizona, USA; 13 School of Education, Psychology and Sports Science, Linneaus University, Kalmar, Sweden; 14 School of Population Health, The University of Western Australia, Crawley, Western Australia, Australia; 15 New Zealand Ministry of Health, Wellington, New Zealand; 16 Valnesfjord Rehabilitation Center, Osterkloft, Norway; 17 Department of Preventive Medicine and Public Health, Tokyo Medical University, Tokyo, Japan; 18 Department of Sports Medicine for Health Promotion, Tokyo Medical University, Tokyo, Japan; 19 Center of Studies of the Physical Fitness Research Center from São Caetano do Sul, CELAFISCS, São Paulo, Brazil; 20 Sport New Zealand (McLean), Wellington, New Zealand; 21 Department of Biosciences and Nutrition at Novum, Unit for Preventive Nutrition, Karolinska Institute, Stockholm, Sweden; 22 Oppegård Municipality, Oppegård, Norway; 23 Department of Kinesiology and Rehabilitation Sciences, Katholic University, Leuven, Belgium; 24 Department of Sport Science, Lithuanian Academy of Physical Education, Kaunas, Lithuania

**Keywords:** Physical activity, Built environment, Neighborhood environment, International, Generalizability, Moderator

## Abstract

**Background:**

Increasing empirical evidence supports associations between neighborhood environments and physical activity. However, since most studies were conducted in a single country, particularly western countries, the generalizability of associations in an international setting is not well understood. The current study examined whether associations between perceived attributes of neighborhood environments and physical activity differed by country.

**Methods:**

Population representative samples from 11 countries on five continents were surveyed using comparable methodologies and measurement instruments. Neighborhood environment × country interactions were tested in logistic regression models with meeting physical activity recommendations as the outcome, adjusted for demographic characteristics. Country-specific associations were reported.

**Results:**

Significant neighborhood environment attribute × country interactions implied some differences across countries in the association of each neighborhood attribute with meeting physical activity recommendations. Across the 11 countries, land-use mix and sidewalks had the most consistent associations with physical activity. Access to public transit, bicycle facilities, and low-cost recreation facilities had some associations with physical activity, but with less consistency across countries. There was little evidence supporting the associations of residential density and crime-related safety with physical activity in most countries.

**Conclusion:**

There is evidence of generalizability for the associations of land use mix, and presence of sidewalks with physical activity. Associations of other neighborhood characteristics with physical activity tended to differ by country. Future studies should include objective measures of neighborhood environments, compare psychometric properties of reports across countries, and use better specified models to further understand the similarities and differences in associations across countries.

## Background

Physical inactivity accounts for a substantial proportion of the global burden of non-communicable diseases [1-3]. Population-level physical activity varies greatly by country [4-6]. The reasons for such variation are not well understood. As postulated by ecological models [7,8], physical activity is affected by multiple levels of influence, including the built and social environments [9,10]. Empirical evidence suggests that neighborhood design features, such as land use mix, are related to physical activity, primarily walking [11-13]. Recreation environments, such as parks and exercise facilities, are associated with leisure-time and overall physical activity [14]. Findings regarding neighborhood traffic, crime, and aesthetics are equivocal [12,15,16].

To date, most studies examining associations between built environments and physical activity were conducted in single countries, primarily the USA and other high-income countries. Review papers have identified this as a limitation and called for more geographic diversity in study locations [14,17,18]. An international comparison approach is important to advancing the theoretical foundation and empirical evidence of the field. Theoretically, most studies on built environments and physical activity are based on ecological models, which postulate cross-level interactions of influence [8,19]. Conceptually, countries represent unique macro-environments as a result of socio-historical and cultural processes [8,20-22]. Attributes of macro-environments are likely to modify the associations between neighborhood environments and physical activity, but this has rarely been tested. Empirically, comparisons of associations across countries provide tests of generalizability in an international setting.

Recently, researchers from the USA, Australia, Belgium, and Sweden conducted studies with comparable designs to examine the association between neighborhood walkability and physical activity [20,23-25]. Most findings from these studies supported similar associations across countries, suggesting evidence of generalizability of some associations, such as the association between objectively measured neighborhood walkability and accelerometry-based physical activity. However, such comparisons should be expanded to a larger geographic area, particularly including lower and middle income countries.

The present study addresses generalizability through country-specific analyses of associations between attributes of neighborhood environments and physical activity. Data were collected from 11 countries on five continents using common methodologies, making it possible to compare associations across countries [6]. Similar patterns of associations indicate evidence of generalizability. Distinctive patterns of associations suggest country as a potential moderator for the association between neighborhood environments and physical activity. We hypothesize that there is generalizability across most countries, in which activity-friendly neighborhood attributes (e.g., mixed land use) are positively associated with physical activity. We expect, however, that the consistency of associations across countries will differ for some attributes of neighborhood environments that may vary widely (e.g., residential density, transit access) or be more subjective (e.g., safety from crime).

## Methods

### Sampling and procedures

The International Prevalence Study was a collaborative international project. The primary aim of the study was to determine nationally representative prevalence of physical activity for international comparisons. Investigators were invited to participate, but needed to demonstrate capacity and agree to follow rigorous protocols to ensure comparability of data collection methods across countries. A description of the research protocols and inclusion criteria was published elsewhere [6]. Of the 24 countries that expressed interest, 20 met the inclusion criteria and conducted data collection. Eleven countries included an environmental survey: Belgium, Brazil, Canada, Colombia, Hong Kong (Special Administrative Region of China), Japan, Lithuania, New Zealand, Norway, Sweden, and the USA. Of these countries, Brazil, Colombia, and Lithuania are upper-middle-income countries and the rest are high-income countries/regions [26]. Informed consent was provided in verbal or written format from all participants and ethics approval was obtained in each participating country.

Sampling, recruitment, survey translation/adaptation, and data collection followed established protocols while allowing for minor modification in local settings (e.g., using random digit dialing or computer-assisted telephone interview) [6]. In each country, the study sample was required to be 18–65 years of age (18–40 in Japan) and representative of the overall population in a country or a significant region within a country (i.e. population of > 1,000,000). Households were randomly selected within each country/region, and individuals within households were selected at random or by most recent birthday. The data collection was conducted in spring or fall 2002/2003 to reduce seasonal variation in physical activity. Questionnaires were either self-administered or administered by interviewers through phone or face-to-face interviews. Current analyses were restricted to participants living in towns or cities with populations ≥30,000 because the environmental measures were not suitable for rural neighborhoods. Demographic characteristics and other descriptive statistics of the analysis sample were presented in a previous paper [27].

### Measures

In all non-English speaking countries, surveys were back-translated to English and approved by investigators before data collection.

#### Environmental attributes

Attributes of neighborhood environments were measured using items from the Physical Activity Neighborhood Environment Survey (PANES) [27,28]. The test-retest reliability of the questionnaire was supported in several countries [28-30]. Each single item of the questionnaire was validated against a relevant multi-item subscale of the abbreviated Neighborhood Environment Walkability Scale (NEWS-A) (Spearman correlations: 0.27 - 0.81) [29]. Neighborhoods were defined as the area within a 10-to 15-minute walk from home. Seven common items were asked in all 11 countries and were used in the current analysis. Participants reported the main type of housing in their neighborhood (e.g., apartment, townhouse, single family home) as a proxy measure for residential density. Having shops and other retail destinations in the neighborhood was used as a marker for land-use mix. The presence of transit stops (e.g. bus stops or train stations) near home was asked because public transportation often involves walking [31]. Questions were asked about the presence of sidewalks, bicycle facilities, and free or low-cost recreation facilities (e.g., parks, public swimming pools) as they provide opportunities for physical activity. Participants reported whether crime in the neighborhood made it unsafe to go on walks at night, as a marker for personal safety. The original response options ranged from 1 (strongly agree) to 4 (strongly disagree) and were recoded as “strongly agree/agree” vs. “disagree/strongly disagree,” with the exception of housing type that was dichotomized to contrast detached single-family homes (i.e., lower residential density) from the rest (higher residential density) [27]. Based on the literature [15,19], we hypothesized that higher residential density, the presence of shops, transit stops, sidewalks, bicycle facilities, low-cost recreation facilities near home, and better personal safety were positively associated with physical activity. We reversed the coding when necessary to reflect the expected direction of associations.

#### Physical activity

The International Physical Activity Questionnaire (IPAQ) short format was used to assess the frequency and duration of past-week walking, moderate-intensity, and vigorous-intensity physical activity that lasted for at least 10 minutes. Questions were designed to measure physical activity across all domains. Evaluation of the short IPAQ in 12 countries concluded that the questionnaire had good one-week test-retest reliability and fair-to-moderate criterion validity when compared against accelerometer total counts [32]. When used to classify achieving physical activity guidelines or not, the short IPAQ was found to have acceptable specificity but low sensitivity [33]. The IPAQ questions were used to determine whether participants met the recommended level of physical activity, defined as 75 minutes/week of vigorous physical activity or 150 minutes of moderate physical activity accumulated in a week through any combination of walking, moderate, or vigorous physical activities [34].

### Data analysis

#### Country-specific analyses

Data from each country were pooled and weighted to account for differential probabilities of sample selection within each country and to improve sample representativeness. Logistic regression was used to examine the association of each environmental variable with meeting physical activity recommendations. To examine whether the association of a neighborhood attribute with physical activity differed by country, a neighborhood attribute × country interaction was included in each model. A significant interaction suggests that the association between an environmental attribute and physical activity was not equivalent across all countries, and therefore country-stratified analyses were warranted. Forest plots were used to display the odds ratios and 95% confidence intervals for associations in each country. All models were adjusted for age and gender as they were the only common demographic variables. We conducted sensitivity analyses by repeating the regression analyses with additional key covariates (educational attainment and car ownership) in countries where these data were available (nine countries collected data on educational attainment, seven countries on car ownership). Statistical analyses were conducted in 2012 using SPSS 19.0 (SPSS Inc.).

#### Post-hoc power analyses

Because statistical power is a common concern in research on environments and physical activity, post-hoc power analyses were conducted to aide interpretation of non-significant associations [35]. Statistical power was calculated based on four key variables: the prevalence rate of the exposure (i.e., the environmental attribute), the prevalence rate of the outcome (i.e., meeting physical activity recommendations), effect size (as measured by odds ratio), and sample size. An association with a significance level at *p*=0.05 was equivalent to the critical value for rejecting the null hypothesis, which was also equivalent to having 0.50 power. Those significant at *p*<0.05 had more than 0.50 power and those non-significant had less than 0.50 power. Statistical power increases with increases in effect sizes and sample sizes and decreases as the prevalence rates of the exposure and outcome deviate from 0.50.

## Results

**Figure 1:**
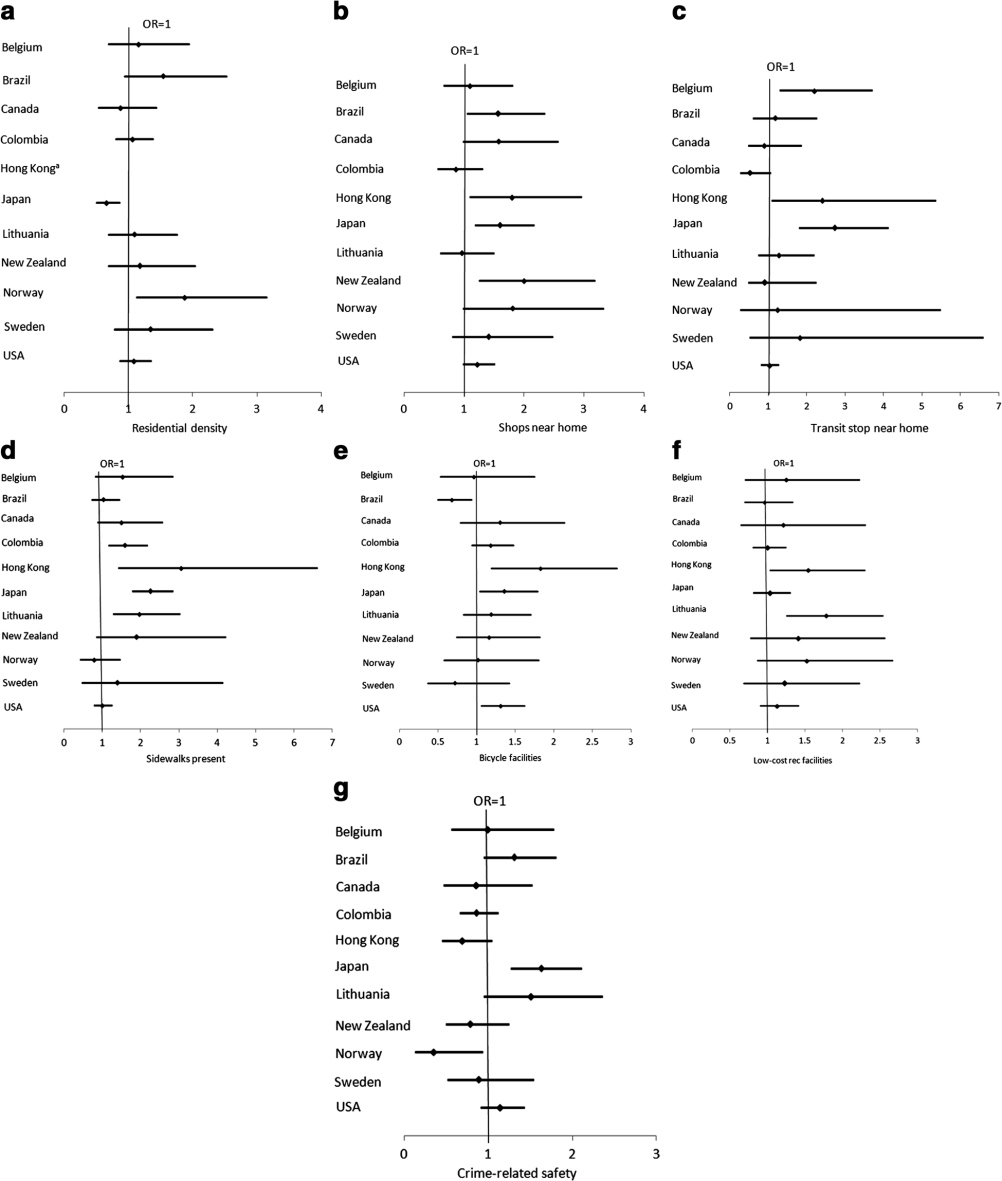
**(1a-1g) Country**-**specific odds ratios for associations between attributes of neighborhood environments and meeting physical activity recommendations.**

Neighborhood attribute × country interactions were significant in all models tested. Therefore, analyses were stratified by country. Country-specific associations are presented in Figure 1.

### Country-specific associations

#### Residential density

Higher residential density was associated with higher odds of meeting physical activity recommendations in Norway; however, the association was in the opposite direction in Japan. Odds ratios in Hong Kong could not be calculated due to the lack of variance in the main housing type (only 3 out of 1100 lived in neighborhoods where the main type of housing was single-family homes).

#### Shops near home

In most countries, the association of having shops near home and physical activity was positive as expected. Associations in Brazil (OR=1.57; 95% CI: 1.05, 2.35; *p*=0.027), Hong Kong (OR=1.80; 95% CI: 1.09, 2.97; *p*=0.023), Japan (OR=1.60; 95% CI: 1.18, 2.17; *p*=0.002), and New Zealand (OR=2.00; 95% CI: 1.26, 3.18; *p*=0.003) reached statistical significance (*p*<0.05). Associations approached significance (0.05<*p*<0.10) in Canada (OR=1.58; 0.98, 2.57; *p*=0.062), Norway (OR=1.81; 95% CI: 0.98, 3.33; *p*=0.058), and the USA (OR=1.22; 95% CI: 0.98, 1.51; *p*=0.079), but the confidence intervals overlapped 1.

#### Transit stop near home

Having public transit stops near home had a positive and significant association with meeting physical activity recommendations in Belgium (OR=2.19; 95% CI: 1.29, 3.72; *p*=0.04), Hong Kong (OR=2.41; 95% CI: 1.09, 5.37; *p*=0.031), and Japan (OR=2.73; 95% CI: 1.80, 4.13; *p*<0.001). In Colombia, the association was near-significant, but in the opposite direction (OR=0.53; 95% CI: 0.27, 1.07; *p*=0.075). The association in Sweden was in the expected direction, but the confidence interval was too wide to make an accurate estimate. Associations in most other countries were close to zero.

#### Sidewalks present

In most countries, having sidewalks present in the neighborhood had a positive association with physical activity. The association was significant in Colombia (OR=1.60; 95% CI: 1.17, 2.19; *p*=0.003), Hong Kong (OR=3.06; 95% CI: 1.42, 6.62; *p*=0.004), Japan (OR=2.26; 95% CI: 1.78, 2.86; *p*<0.001), and Lithuania (OR=1.97; 95% CI: 1.28, 3.03; *p*=0.002). In Canada, Norway, Sweden, and the USA, the association was in the expected direction, but the confidence intervals overlapped OR=1.

#### Bicycle facilities

Having bicycling facilities present in the neighborhood had a significant association with higher odds of meeting physical activity recommendations in Hong Kong (OR=1.83; 95% CI: 1.19, 2.82; *p*=0.006), Japan (OR=1.36; 95% CI: 1.04, 1.79; *p*=0.026), and the USA (OR=1.31; 95% CI: 1.06, 1.62; *p*=0.013). This association, however, was inverse and significant in Brazil (OR=0.68; 95% CI: 0.50, 0.93; *p*=0.014).

#### Low-cost recreation facilities

The presence of free or low-cost recreation facilities in the neighborhood was only significantly associated with higher odds of meeting physical activity recommendations in Hong Kong (OR=1.54; 95% CI: 1.03, 2.30; *p*=0.036) and Lithuania (OR=1.78; 95% CI: 1.25, 2.54; *p*=0.002). Associations in most countries had wide confidence intervals that overlapped 1.

#### Safety from crime

Crime-related safety had an inconsistent association with physical activity across countries. The association was positive and significant in Japan (OR=1.63; 95% CI: 1.27, 2.11; *p*<0.001), positive and approaching significance in Brazil (OR=1.31; 95% CI: 0.95, 1.81; *p*=0.09) and Lithuania (OR=1.50; 95% CI: 0.95, 2.36: *p*=0.08), inverse and significant in Norway (OR=0.35; 95% CI:0.13, 9.40; *p*=0.037), and inverse and approaching significance in Hong Kong (OR=0.69; 95% CI: 0.45, 1.05; *p*=0.08).

### Sensitivity analyses

Results from sensitivity analyses suggested that by including educational attainment (as a marker for socioeconomic status) in the nine countries where such data were collected, the magnitude of association changed only by 0 to 3%. By including car ownership as an additional covariate in the seven countries with existing data, the magnitude of the current association changed by 0 to 9%. This suggests that including educational attainment and car ownership in the model was unlikely to lead to sizable difference in the results.

### Post-hoc power analyses

Table 1 shows results from post-hoc power analyses. The columns Prx [Pr(x=1)] present the prevalence rates of exposures (i.e., the presence of an environmental attribute) in each country. The columns Pry [Pr(y=1 ׀ x=0)] refer to the probability of having the outcome (i.e., meeting physical activity recommendations) given that the environmental attribute was not present. The column “n” shows the actual sample size in each country. Numbers in bold indicate tests of associations with more than 0.50 power. Underlined numbers signify the associations that are close to statistical significance (0.05<*p*<0.1) with statistical power that is slightly below 0.50.

**Table 1:** **Weighted sample characteristics of participants by country** (**2002**–**2003**)

	**Belgium (n**=**348)**	**Brazil (n**=**876)**	**Canada (n**=**634)**	**Colombia (n**=**2692)**	**Hong Kong (n**=**1100)**	**Japan (n**=**1221)**	**Lithuania (n**=**1245)**	**New Zealand (n**=**797)**	**Norway (n**=**500)**	**Sweden (n**=**440)**	**USA (n**=**2560)**
Age: Mean (SD)	42.3 (12.0)	35.6 (12.4)	39.3 (12.5)	36.7 (12.5)	39.5 (10.8)	32.2 (5.5)	37.5 (13.1)	39.0 (12.7)	38.2 (12.4)	38.8 (12.9)	40.2 (12.4)
Female (%)	44.4	49.5	44.8	51.5	53.1	30.9	52.8	57.9	47.9	50.4	57.6
Meeting physical activity recommendations	75.9	70.9	87.6	86.6	89.1	55.9	88.6	88.9	86.6	83.2	82.2
Environmental characteristics (%)											
High residential density	66.4	12.0	39.8	78.9	99.7	70.8	84.3	24.4	58.1	70.4	40.2
Shops near home	63.1	85.0	67.1	92.2	88.4	82.8	82.2	73.4	83.9	77.0	59.0
Transit stops	74.6	94.8	85.2	95.9	96.4	90.6	90.6	91.4	97.4	97.1	68.4
Sidewalks present	83.5	24.8	79.7	88.5	96.9	58.1	86.3	94.5	76.9	95.4	74.6
Bicycle facilities	78.1	33.4	68.0	40.6	37.2	24.5	46.9	45.4	72.3	79.9	56.5
Recreation facilities	78.2	28.1	86.0	50.9	72.9	59.4	53.8	87.2	76.4	78.8	69.1
Crime-related safety	75.8	34.8	79.0	24.2	63.7	67.3	25.0	57.3	84.8	60.8	66.8

## Discussion

This study examined whether associations between neighborhood environments and physical activity differed by country. Based on representative samples from 11 countries, we used standardized methodologies that allowed for cross-country comparisons. We found that the associations of physical activity with land use mix and sidewalks were relatively consistent across countries, suggesting evidence of generalizability. However, associations with other neighborhood characteristics tended to vary more across countries.

The association of perceived land use mix (as measured by shops near home) with physical activity was significant in 4 countries and approached significance in another 3 countries. This suggests that mixed land use is an important attribute that is likely to facilitate physical activity in a wide range of countries. This finding echoed that from a recent meta-analysis where land-use diversity and access were the strongest correlates of walking [13].

The presence of sidewalks was significantly associated with physical activity in 4 countries. In several other countries (e.g., Belgium, Canada, New Zealand), the association was in the same direction and of similar magnitude but without reaching statistical significance. The insufficient power was due to combined factors of low variance in the independent or dependent variable and small sample sizes in these countries. Previous reviews concluded that the presence of pedestrian infrastructure was positively associated with overall physical activity and walking [12,36]. The present study extended current knowledge by providing evidence of generalizability from a wider range of countries.

The presence of bicycle facilities was significantly associated with physical activity in Hong Kong, Japan, and the USA. However, the mechanism for this association is unknown because bicycle use was not measured. Interestingly, in this study, the presence of bicycle facilities was not predictive of meeting physical activity recommendations in European countries where bicycling is more common [37]. Generally speaking, Western European countries have good infrastructure, policies, and social norms for bicycling [38]. Therefore the current measure of bicycle facilities could not capture sufficient variance.

Public transit access has been less frequently examined as a correlate of physical activity. In the current study, the presence of transit stops was significantly associated with physical activity in Belgium, Hong Kong, and Japan. The association was strong in these three countries (OR>2), but weak in most other countries. One potential explanation is that because transportation mode was not measured, it is unknown how much physical activity in each country was attributable to public transit use. Particularly in countries like the USA where public transit use is rare [37], the contribution of transit use to overall physical activity could be trivial. Another possible explanation for the lack of association is that in most countries access to public transit was highly prevalent (more than 90% of participants reported having a transit stop near home in eight countries). The lack of variance could result in underestimated associations. To enrich current data and improve variance, future studies should examine additional aspects of public transit, such as pricing, frequency, and quality of service [39].

Only a small number of associations that involved access to recreation facilities were significant. However, most non-significant associations were in the expected direction. Because the effect sizes were small, the power for detecting significant associations was limited. This finding suggests that the presence of parks and other recreation facilities in the neighborhood could be a generalizable but weak correlate of physical activity in most countries.

Both residential density and land-use mix are key components of neighborhood walkability [40]. In this study, however, there was little support for the association between residential density and physical activity. This may be because the measure of residential density was only a crude proxy. This may also suggest that land-use mix had more predictive validity and may be a more important component of walkability than residential density. A previous meta-analysis of built environments and travel behavior had similar findings and suggested that density could be an intermediate variable that influenced travel behavior through other variables such as land use mix [13].

Crime is a frequently cited barrier to physical activity, but its association with physical activity has been inconsistent [41]. The current analyses revealed similarly inconsistent findings. The association between crime-related safety and physical activity is complex because different types of crime, timing and context (e.g., day-time vs. night-time), emotional responses, and coping strategies (e.g., constrained vs. protective behavior) may affect physical activity differently. Furthermore, people from different countries and cultures may have different perceptions about safety and cope with unsafe neighborhoods differently. Also, the association between crime and physical activity might be confounded by residential density, a component of walkability. Future studies should test more complex models, compare psychometric properties of crime/safety measures across countries, and adjust for potential environmental confounders.

Previous pooled analyses of the 11 countries found that five of the seven environmental correlates were significant, including shops near home, transit stops, sidewalk present, bicycle facilities, and low-cost recreation facilities [27]. The higher percentage of significant associations compared to current analyses was due to more power as a result of a larger sample size and more variability in data. However, a potential drawback of such pooled analysis is that it averages effects that could be different across countries. For example, access to transit stops was significant in the pooled analysis; however, in country-specific analyses this association was close to zero in most countries. A more comprehensive overview of the evidence base should consider both the overall patterns of associations in internationally pooled analyses and tests of generalizability in country-specific analyses.

### Limitations

The geographic variation, population representativeness, standardized methodology and measures provided a rare opportunity for comparisons across countries. However, this study had some limitations. First, physical activity was a dichotomous variable and was measured only by the IPAQ short form, which has often led to considerable overestimation compared to objective measures [42]. Furthermore, it is unknown whether the degree of possible overestimation of physical activity was different across countries. For example, researchers found that in Latin America people tend to over-report household and occupational physical activity [43]. Such systematic biases are likely to affect associations between neighborhood environments and total physical activity. The IPAQ short form also does not differentiate between domains (e.g., transport, leisure-time) or types of physical activity (e.g., bicycling, public transit use). Therefore, it was impossible to test more specific hypotheses. Second, standardized instruments could not take into account country-specific situations. For example, the PANES question about housing types resulted in almost zero variance in Hong Kong, making tests of association impossible. Future studies should consider balancing the “trade-off” between using standardized instruments to improve comparability and using specific instruments to capture uniqueness within certain geographic areas. It is also important to note that even with standardized instruments, people from different countries are likely to perceive environments, interpret questions, and provide answers differently. This might be particularly relevant to questions regarding personal safety and aesthetics. Third, because neighborhood environments were measured by one’s perception only, we cannot exclude the possibility that those who were more active were more observant of their neighborhoods and were more likely to report activity-friendly features, such as shops near home. Fourth, questions were only asked about the neighborhoods around home even though not all people would spend most of their time in their neighborhood. Therefore, future studies should take into account non-home neighborhood environments in addition to home neighborhood environments [44]. Fifth, although study samples were intended to be nationally or regionally representative, response rates varied across countries. This might imply different sampling biases across countries. However, sample representativeness is generally a bigger concern for prevalence studies than association studies. Sixth, some key variables that might modify or confound country-specific associations were not collected, such as climate. Last but not least, several countries had relatively small sample sizes and/or skewed data distribution that led to under-powered statistical tests. Therefore, an association of similar magnitude could be significant in one country, but non-significant in another. As statistical power is a major concern in environmental studies, it is important to consider different factors that affect statistical power. Future studies might use Table 2 as a tool for power calculation and results interpretation.

**Table 2:** **Post**-**hoc power analyses for logistic regression in 11 countries**

	**Dependent variable: meeting physical activity recommendations**																					
		**Residential density**			**Shops near home**			**Transit stops near home**			**Sidewalks present**			**Bicycle facilities**			**Low-cost rec facilities**			**Crime-related safety**		
	**n**	**Prx**	**Pry**	**Power**	**Prx**	**Pry**	**Power**	**Prx**	**Pry**	**Power**	**Prx**	**Pry**	**Power**	**Prx**	**Pry**	**Power**	**Prx**	**Pry**	**Power**	**Prx**	**Pry**	**Power**
Belgium	348	0.66	0.74	0.08	0.63	0.74	0.06	0.75	0.64	**0.82**	0.84	0.69	0.26	0.78	0.76	0.02	0.78	0.72	0.12	0.76	0.76	0.03
Brazil	876	0.12	0.70	0.41	0.85	0.63	**0.62**	0.95	0.65	0.08	0.25	0.71	0.04	0.33	0.74	**0.70**	0.28	0.71	0.04	0.35	0.69	0.40
Canada	634	0.40	0.87	0.08	0.67	0.83	0.47	0.85	0.88	0.05	0.80	0.83	0.33	0.68	0.85	0.19	0.86	0.84	0.09	0.79	0.88	0.10
Colombia	2692	0.79	0.86	0.06	0.92	0.88	0.10	0.96	0.92	0.42	0.89	0.81	**0.84**	0.41	0.86	0.29	0.51	0.86	0.03	0.24	0.86	0.23
Hong Kong	1100	1.00	NA^a^	NA	0.88	0.83	**0.64**	0.97	0.78	**0.53**	0.97	0.74	**0.78**	0.37	0.87	**0.79**	0.73	0.86	**0.54**	0.65	0.91	0.42
Japan	1221	0.71	0.63	**0.90**	0.83	0.45	**0.87**	0.91	0.32	**0.99**	0.58	0.44	**1.00**	0.25	0.54	**0.63**	0.59	0.56	0.04	0.67	0.49	**0.98**
Lithuania	1245	0.84	0.87	0.06	0.82	0.89	0.04	0.91	0.86	0.14	0.86	0.81	**0.88**	0.47	0.87	0.17	0.54	0.85	**0.88**	0.25	0.88	0.43
New Zealand	797	0.24	0.88	0.09	0.73	0.83	**0.85**	0.91	0.88	0.04	0.95	0.82	0.32	0.45	0.88	0.10	0.87	0.85	0.21	0.57	0.89	0.19
Norway	500	0.58	0.81	**0.69**	0.84	0.79	0.47	0.97	0.85	0.05	0.77	0.88	0.11	0.72	0.86	0.03	0.76	0.82	0.32	0.85	0.94	**0.55**
Sweden	440	0.70	0.80	0.20	0.77	0.78	0.23	0.97	0.77	0.15	0.95	0.80	0.09	0.80	0.87	0.16	0.79	0.81	0.10	0.61	0.85	0.06
USA	2560	0.40	0.84	0.10	0.59	0.83	0.44	0.68	0.84	0.04	0.75	0.84	0.03	0.57	0.82	**0.70**	0.69	0.83	0.18	0.67	0.83	0.21

Prx: Pr(x=1), the probability of having a neighborhood attribute (e.g., having shops near home).

Pry: Pr(y=1׀x=0), the probability of having the outcome (meeting physical activity recommendations) given that the neighborhood attribute is not present.

Bolded numbers: statistical tests with >0.50 power based on post-hoc power analyses for logistic regression.

Underlined numbers: associations that are close to statistical significance (0.05<p<0.1) with statistical power that is slightly below 0.50.

^a^ Could not be calculated due to the lack of variance in residential density.

## Conclusions

Using population representative data from 11 countries this study provided evidence for the generalizability of the associations between neighborhood land use mix, sidewalks and physical activity. Associations of public transit access, bicycle facilities, and recreation facilities with physical activity were more variable across countries. There was little support for associations of physical activity with residential density and crime-related safety. Future studies should continue to examine the associations between neighborhood environments and physical activity in an international setting to better understand the similarities and differences across countries. Priority areas of improvement may include adopting objective measures and specific reports of physical activity by domain, testing better specified models, including a broader range of neighborhood environments, and examining differential response bias to improve comparability of survey instruments across countries.

## Abbreviations

IPAQ: International Physical Activity Questionnaire; NEWS: Neighborhood Environment Walkability Scale; PANES: Physical Activity Neighborhood Environment Survey; USA: United States of America.

## Competing interests

The authors declare that they have no competing interests.

## Authors’ contributions

DD and JFS conceptualized the research idea. DD analyzed the data and drafted the paper. DD, MAA, JFS, GJN, MFH, CDC, CRH contributed to the analysis and interpretation of data. AEB, CRH, HRB, MH, CLC, LFG, IDB, DJM, BEA, PB, FCB, HC, LKH, SI, NM, SM, VM, GM, MS, HT, JL, VV contributed to the conceptualization and design of the original study and the acquisition of data. All authors were involved in revising the manuscript for important intellectual content. All have given final approval of the version currently submitted to the International Journal of Behavioral Nutrition and Physical Activity.
